# *SWAP-MEAT Athlete* (study with appetizing plant-food, meat eating alternatives trial) – investigating the impact of three different diets on recreational athletic performance: a randomized crossover trial

**DOI:** 10.1186/s12937-022-00820-x

**Published:** 2022-11-16

**Authors:** Aubrey K. Roberts, Vincent Busque, Jennifer L. Robinson, Matthew J. Landry, Christopher D. Gardner

**Affiliations:** Stanford Prevention Research Center, Stanford University School of Medicine, Stanford, CA USA

**Keywords:** Plant-based diet, Whole food plant-based diet, Plant-based meat alternatives, Athletic performance, Sports performance

## Abstract

**Background:**

Plant-based diets are known to be beneficial for cardiovascular health and promote environmental sustainability. However, many athletes avoid plant-based diets due to concerns of protein inadequacy.

**Objectives:**

To investigate the impact of two predominately plant-based diets—whole food plant-based (WFPB) and plant-based meat alternatives (PBMA)—vs. an omnivorous diet, favoring red meat and poultry (Animal), on endurance and muscular strength.

**Methods:**

12 recreational runners and 12 resistance trainers were assigned to three diets—WFPB, PBMA, and Animal—for 4 weeks each, in random order. Primary outcomes for runners (12-minute timed run) and resistance trainers (composite machine strength) were collected at baseline and after diets, along with secondary performance outcomes and dietary data.

**Results:**

22 recreational athletes completed the study (age: 26.2 ± 4.4 years; sex: 10 female, 12 male; BMI: 23.1 ± 2.4 kg/m^2^). Mean differences in 12-minute timed run – WFPB vs. Animal (− 23.4 m; 95% CI: − 107 to 60.0 m) and PBMA vs. Animal (− 2.9 m; 95% CI: − 119 to 113 m) – were not significant. Mean percent differences in composite machine strength – WFPB vs. Animal (− 2.7%; 95% CI: − 5.8 to 0.4% and PBMA vs. Animal (− 0.7%; 95% CI: − 3.5 to 2.2%) – were not significant. Average protein intake for all diets met International Society for Sports Nutrition recommendations.

**Conclusions:**

Our findings suggest recreational athletes can maintain athletic performance on both an omnivorous diet and two diets that are predominately plant-based.

**Trial registration:**

NCT05472701. Retrospectively registered.

**Supplementary Information:**

The online version contains supplementary material available at 10.1186/s12937-022-00820-x.

## Introduction

Plant-based diets—dietary patterns focusing on vegetables, legumes, fruits, nuts and seeds, and whole grains—are associated with improved cardiovascular outcomes and environmental sustainability compared to omnivorous diets [1–3]. Plant-based diets are high in fiber and low in saturated fat which leads to improvements in cardiovascular disease (CVD) risk factors, including lower LDL cholesterol, blood pressure, and body mass index [4]. Production of natural, plant-based foods also reduces greenhouse gas (GHG) emissions, pollution, and land and water usage compared to animal products [5]. The American Heart Association both recognizes these environmental benefits and recommends that protein intake should primarily come from natural plant sources to promote cardiovascular health [6]. The International Panel on Climate Change also recognizes consumption of plant-based diets as a key mitigation strategy for climate change [7].

Despite the benefits of plant-based diets, many athletes are reluctant to adopt them—fearing they will not consume enough protein and/or calories to build strength, recover, and perform [8]. However, medical professionals recognize that plant-based diets can meet the nutritional needs of athletes, and most Americans exceed daily protein recommendations [9, 10]. Critics are also concerned that “protein quality” is compromised on a plant-based diet. However, all 20 amino acids, including the 9 essential amino acids, can be obtained by consuming a well-balanced variety of plant foods [10, 11]. By overemphasizing protein intake, athletes can also neglect to consume enough carbohydrates, the body’s primary energy source, to fuel activity [12].

Interestingly, plant-based diets are high in carbohydrates which could enhance endurance performance by facilitating glycogen storage [13]. Plant-based diets are also associated with a reduction in inflammatory markers such as C-reactive protein [14]. High fiber and low fat content may promote loss of body fat and increase lean muscle mass [13]. In addition, the low cholesterol content of plant-based diets reduces blood viscosity, promotes arterial flexibility, and increases tissue oxygenation [13]. Athletes may be missing benefits of plant-based diets–including adequate protein and carbohydrate consumption, reduced inflammation, higher proportions of lean muscle mass, and better cardiovascular health–by overemphasizing animal products and protein intake.

Existing literature suggests consumption of plant-based vs. omnivorous diets does not lead to significant differences in athletic performance, but these studies are often cross-sectional which limits causal inference [15, 16]. Existing randomized crossover trials are sparse and have small sample sizes (*n* < 10), investigate only one type of athletic performance (endurance or muscular strength), or exclusively focus on elite male athletes [17, 18]. Most research focuses on traditional definitions of plant-based diets (i.e., lacto-ovo-vegetarian, vegan), without exploring how consumption of emerging “plant-based meat alternative” products impact athletic performance. Plant-based meat alternatives—such as plant-based burgers, patties, or sausages—are increasing in popularity and differ from traditional plant-based foods in that they are more convenient, processed, and designed to mimic animal meat. Evidence regarding cardiovascular benefits and environmental sustainability of plant-based meat alternative diets is mixed due to the high sodium and saturated content of the products, as well as their processed nature. However, recent reviews generally suggest improvements in these metrics when compared to animal meat, although benefits are attenuated when compared to natural plant-based foods [19].

The goal of the present study is to compare the impact of two predominately plant-based diets—whole food plant-based (WFPB) and plant-based meat alternatives (PBMA)—to an omnivorous diet (Animal) on endurance performance and muscular strength in recreational athletes. We define WFPB as a holistic diet pattern that emphasizes intake of natural, plant-based proteins and minimizes intake of dairy, egg, and processed food. PBMA emphasizes intake of plant-based meat alternatives as the primary protein source, whereas Animal emphasizes red meat and poultry as the primary protein source—permitting fish consumption only once per week. This study will also gather data on dietary composition and dietary adherence, and thereafter assess the feasibility of a larger randomized crossover trial.

## Methods

The study protocol was approved on April 2, 2021 (Protocol ID #59905). All participants provided written informed consent.

### Study design

*SWAP-MEAT Athlete* (Study With Appetizing Plant-Food - Meat Eating Alternatives Trial) builds upon the *SWAP-MEAT* randomized crossover trial that investigated CVD risk factors in adults after 8-week PBMA and Animal diets [20]. *SWAP-MEAT Athlete*, a novel randomized crossover trial, explores athletic performance in 12 runners and 12 resistance trainers completing three 4-week diets—WFPB, PBMA, and Animal—in a random order, without washout periods. Consumption of two servings of diet-specific protein sources was required each day. Examples included quinoa, lentils, and beans (WFPB), Beyond Meat, Impossible Foods, or Gardein (PBMA), and poultry and red meat (Animal) (Supplementary Table 1). Participants were to maintain a consistent physical activity routine throughout the study. Each week, participants completed physical activity logs, food adherence surveys, and logged dietary intake. Athletic performance was measured at baseline and after diets (week 4, 8, 12). Primary outcomes were Cooper 12-minute timed run (runners) and machine composite strength (resistance trainers). Secondary outcomes included estimated VO_2_ max (runners) and maximum push-up and pull-up tests (resistance trainers). Rate of perceived exertion, anthropometrics, and diet satisfaction data were obtained.

### Study participants

Participants were generally healthy, recreational runners and resistance trainers. Inclusion criteria included the following: 1) between 18 and 35 years old; 2) at least 1 year of consistent training (three to four times a week); 3) omnivorous diet for at least 6 months; and 4) body mass index (BMI) 18.5 - 30 kg/m^2^. Participants were excluded for the following: 1) nutrient intolerances, eating disorder; 2) chronic disease, musculoskeletal injuries, or pregnancy; or 3) intention to change training volume or intensity. Runner and resistance trainer groups were recruited so that each had 50% females and 50% males.

### Run-in phase

Participants completed a 2 week run-in phase for familiarization with logging and equipment before beginning diets. Runners wore Garmin GPS watches during weekly runs to estimate VO_2_ max.

### Randomization

Participants were randomized to one of six possible diet orders with the three arms of WFPB, PBMA, and Animal. Randomization was performed by an independent researcher in two blocks of six for each athlete type (runner, resistance trainer). A random number generator was used to assign the six diet orders within each block, and runners and resistance trainers were assigned to blocks in the order they completed baseline athletic field tests.

### Dietary intervention and assessment

All diets (WFPB, PBMA, Animal) lasted 4 weeks each. During WFPB, participants consumed at least two meals consisting of vegetables, legumes, fruits, nuts and seeds, and whole grains each day. Common meals consisted of protein sources including quinoa, beans, and tofu, among others (Supplementary Table 1). Intake of processed food, eggs, and dairy was minimized—this diet was designed to be based around natural plant foods, with rare to occasional intake of any of these other products. No consumption of animal meat, plant-based meat alternatives, or fish was allowed.

During PBMA, participants were to consume at least two servings of plant-based meat alternative protein sources per day. Common protein sources included Beyond Burger, Impossible Burger, and Gardein (Supplementary Table 1). No consumption of animal meat was allowed. Fish consumption was allowed once per week.

During Animal, participants were to consume at least two servings of animal meat per day. Animal meat was to be consumed predominately in the form of red meat or poultry (Supplementary Table 1). Fish consumption was allowed once per week.

For Animal and PBMA, with the exception of protein sources, participants were instructed to match all other food intake as closely as possible (i.e. prepare a burger—animal meat or plant-based meat alternative—with the same hamburger bun, toppings, and condiments). PBMA was designed to be a more convenient version of a plant-based diet that focused on eliminating animal meat and swapping plant-based meat alternatives into meals. Animal products such as dairy and egg could be consumed on PBMA and Animal, but these were auxiliary only – not the primary protein source and consumption was not to be increased. Participants received $100 per diet phase and purchased their own food.

Participants logged their complete dietary intake for 3 days per week (two weekdays, one weekend day) using the tracking application Cronometer (Cronometer Pro, Nutrition Tracking Software for Professionals; https://cronometer.com/pro). Additionally, participants completed food adherence surveys to quantify diet adherence in servings of diet-specific protein consumed per week. The research team monitored all weekly logs and surveys and provided encouragement and positive reinforcment via email and phone communication.

### Physical activity

Participants engaged in running or resistance training at least 3 to 4 days per week for at least 30 minutes. Participants were to maintain a similar duration, intensity, and frequency of workouts each week. Participants completed weekly physical activity logs to monitor adherence to consistent training.

### Athletic field tests

Participants completed athletic field tests at baseline and after diets (week 4, 8, 12). All athletic field tests were conducted remotely during the COVID-19 pandemic. Participants were to perform all athletic field tests under similar conditions: consistent time of day, food/drinks prior, warm-up, equipment and location. Participants were to minimize vigorous exercise before, avoid external distractions like music, and complete athletic field tests within a four day window.

Runners completed the Cooper 12-minute timed run, reporting total distance covered after a 12 minute run at maximal effort [21]. Participants ran on a 400 m track, or a consistent, flat route with their Garmin GPS watch (Garmin Forerunner 235). Runners reported estimated VO_2_ max from watches based on training data from the last 4 weeks. Garmin’s VO_2_ algorithm is 95% accurate (error < 3.5 mL/kg/min) based on an analysis of 2690 runs from 79 runners over 6-9 months [22].

Resistance trainers completed athletic field tests on two days. First, resistance trainers recorded the maximum number of push-ups and pull-ups they completed before exhaustion, with 10-15 minutes rest between tests. These measures of muscular endurance have been used with recreational athletes [23]. Participants used a resistance band (Vivi Life Fitness Resistance Bands) for assistance with pull-ups throughout the study if unable to complete 10 pull-ups at baseline.

After 48 hours, resistance trainers completed 3-rep maximum tests for machine-based exercises (chest press, leg press, lat pull-down), with 10-15 minute rest between tests. Participants recorded the maximum number of kilograms they lifted for three repetitions, no more or no less. Multiple rep max tests have been used with recreational athletes, and 48 hours between tests of similar muscle groups allows for full recovery [24, 25]. Machine-based exercises were compiled into a machine composite strength score where we display a raw, summed composite number (kg) and the average change (%) across machine exercises for an individual.

### Additional measures: RPE, anthropometrics, diet acceptability

Participants used the Borg Rating of Perceived Exertion (RPE) Scale, ranging from 6 (no exertion at all) to 20 (maximal exertion), to rate perception of physical exertion [26]. This was used to gauge effort during athletic field tests and gather session RPE (sRPE) for workouts.

Body weight and body fat percentage were collected with bioelectrical impedance scales (RENPHO Smart Body Fat Scale) on mornings of athletic field tests. Participants used the Food Acceptability Questionnaire to rate diet satisfaction on a 7-point Likert scale before athletic field tests, with higher scores indicating higher satisfaction [27].

### Statistical analysis

Participant baseline characteristics are presented as means ± standard deviation (SD) or percentages. One way repeated-measures ANOVA were used to test for differences in nutrient intake between diets. If significant differences were observed, post hoc analyses were conducted using paired t-tests. Primary and secondary outcomes are expressed as means ± SD or mean differences with 95% confidence intervals. An alpha of .05 was used to indicate statistical significance. Data were analyzed using R version 4.1.0 (R Foundation for Statistical Computing; May 18, 2021). Sample size was based on available resources.

We peformed an ad hoc power analysis for a paired t-test with a 5% significance level. With 11 runners, we had 80% power to detect a mean difference of 140 m in distance covered for WFPB or PBMA vs. Animal, assuming a SD of 150 m. With 11 resistance trainers, the trial had 80% power to detect a mean difference of 4.2% in machine composite strength, assuming a SD of 4.5%.

## Results

### Participant population

**Fig. 1:**
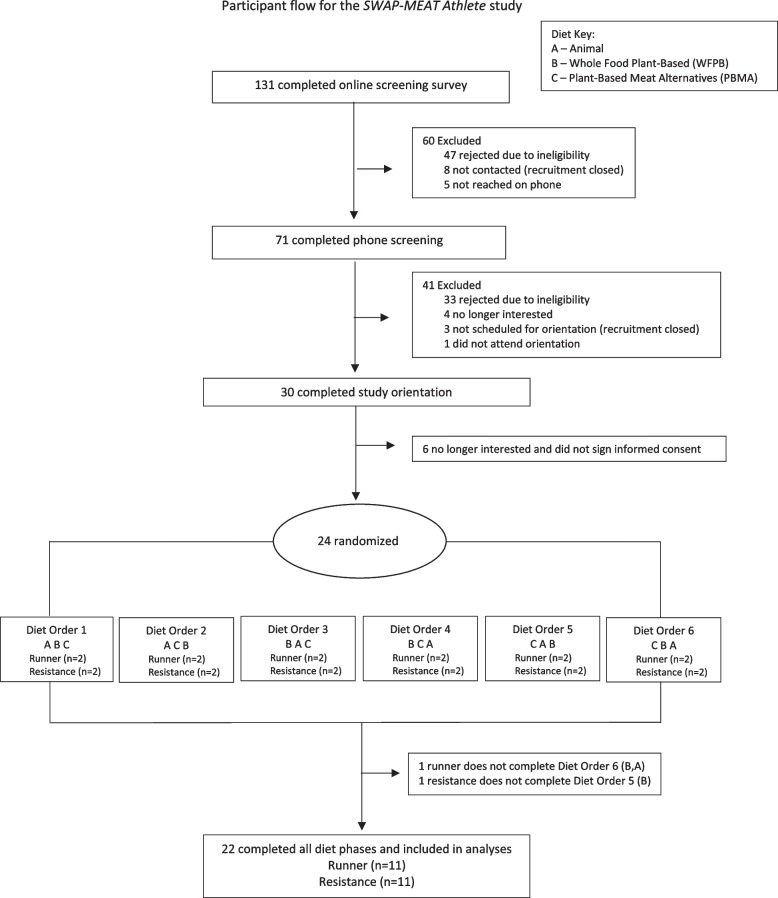
Participant flow for the SWAP-MEAT athlete study

Figure 1 shows participant flow through the study. 24 participants were randomly assigned to one of six diet orders, with two runners and two resistance trainers per diet order. Two participants dropped out due to unanticipated work commitments and were excluded from analysis.

Table 1 presents baseline participant demographics, anthropometrics, and physical activity status. Participants were 26.6 ± 4.4 (mean ± SD) years old, with an average BMI of 23.1 ± 2.4 kg/m^2^. They reported running or lifting 4.4 ± 1.0 days or 220 ± 61 minutes per week on average. Participants were experienced athletes, engaging in running or resistance training for an average of 6.0 ± 4.0 years before the study.

**Table 1:** Participant demographics and baseline characteristics (n, %, or mean ± SD)

	Runners	Resistance Trainers	All
Sex, n			
Female	5	5	10
Male	6	6	12
Age, y	26.2 ± 4.6	26.9 ± 4.3	26.6 ± 4.4
Race/ethnicity, %			
Asian	45.5	36.4	40.9
Hispanic/Latino	36.4	18.2	27.3
Non-Hispanic white	9.1	36.4	22.7
Other	9.1	9.1	9.1
Weight, kg			
Female	61.0 ± 8.9	54.4 ± 4.3	57.7 ± 7.4
Male	67.6 ± 8.6	74.6 ± 10.8	71.1 ± 10.0
Both Sexes	64.6 ± 9.0	65.5 ± 13.2	65.0 ± 11.1
BMI, kg/m^2^			
Female	22.4 ± 1.7	21.5 ± 0.7	22.0 ± 1.3
Male	23.3 ± 2.8	24.7 ± 2.7	24.0 ± 2.7
Both Sexes	22.9 ± 2.3	23.3 ± 2.6	23.1 ± 2.4
Baseline Athletic Traits			
Days run or lift per week, n	4.8 ± 0.9	4.0 ± 1.1	4.4 ± 1.0
Years run or lift, n	5.2 ± 3.9	6.9 ± 4.2	6.0 ± 4.0
Minutes run or lift per week, n	224 ± 66	217 ± 58	220 ± 61
Miles run per week, n	25.5 ± 11.4		

### Dietary intake and adherence

Dietary intake data from Cronometer are presented in Table 2. Participants reported similar energy intake across diets. However, carbohydrates, fiber, protein, and cholesterol were significantly different between all diets; intake of these nutrients existed as a gradient across diets with PBMA as the intermediate. Carbohydrates and fiber were highest for WFPB and lowest for Animal, with PBMA as the intermediate. Protein and cholesterol were lowest for WFPB and highest for Animal, with PBMA as the intermediate. Saturated fat and sodium were lower in WFPB compared to Animal and PBMA. Adherence to 14 servings/week of diet-specific protein source was high, with participants reporting 14.1 ± 3.6, 12.2 ± 2.4, and 13.3 ± 2.5 servings/week in food adherence surveys during WFPB, PBMA, and Animal respectively.

**Table 2:** Summary of dietary intake and adherence^1^

Dietary Intake	WFPB	PBMA	Animal	ANOVA
	Mean ± SD	Mean ± SD	Mean ± SD	*p*-value^2^
Nutrients				
Energy (Kcal)	1903 ± 412	1875 ± 375	1913 ± 421	0.63
Protein (g)	72 ± 13^a^	85 ± 20^b^	107 ± 27^c^	< 0.001^***^
Carbs (g)	259 ± 67^a^	222 ± 58^b^	205 ± 62^c^	< 0.001^***^
Sugars (g)	64 ± 24	61 ± 21	57 ± 24	0.16
Fat (g)	69 ± 19^a^	77 ± 21^b^	75 ± 22^ab^	0.05^*^
Saturated Fat (g)	17 ± 6^a^	23 ± 7^b^	23 ± 8^b^	< 0.001^***^
Unsaturated Fat (g)	52 ± 14	53 ± 16	52 ± 15	0.74
Fiber (g)	40 ± 12^a^	31 ± 10^b^	25 ± 11^c^	< 0.001^***^
Sodium (mg)	2141 ± 750^a^	2712 ± 874^b^	2598 ± 913^b^	0.004^**^
Iron (mg)	18 ± 7^a^	19 ± 6^a^	15 ± 7^b^	< 0.001^***^
Calcium (mg)	860 ± 189	804 ± 230	752 ± 296	0.17
Dietary Cholesterol (mg)	66 ± 75^a^	129 ± 110^b^	313 ± 92^c^	< 0.001^***^

^1^Nutrient intake data from Cronometer where participants logged an average of 15-16 complete days for all diet phases (12 days were required per diet phase). Range of complete days logged was 9-28 days for diet phases

^2^Means with different superscripts were significantly different from each other after post hoc paired t-tests. Means sharing the share superscript are not significantly different.

significance level *p < 0.05, ** *p* < 0.01, ***p < 0.001

**Fig. 2:**
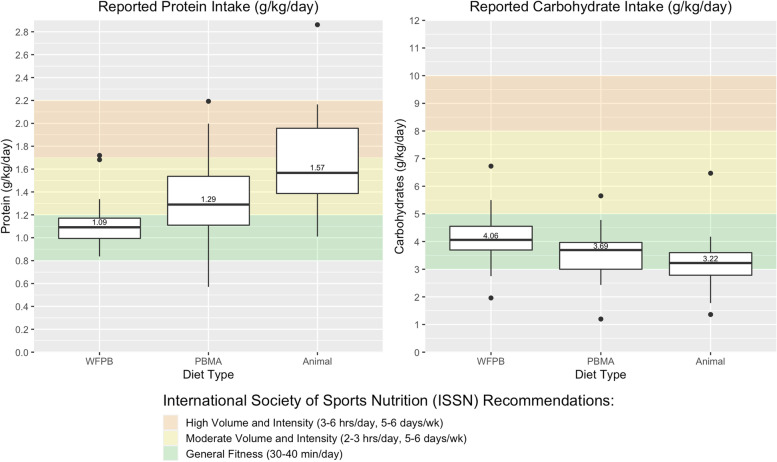
Comparison of Macronutrient Intake (g/kg/day) to International Society for Sports Nutrition (ISSN) recommendations. ISSN recommendations for athletes of general fitness, moderate, and high volume and intensity correspond to protein intake of 0.8-1.2 g/kg/day, 1.2-2.0 g/kg/day, and 1.7-2.2 g/kg/day and carbohydrate intake of 3-5 g/kg/day, 5-8 g/kg/day, and 8-10 g/kg/day respectively [18]. Note that moderate and high volume and intensity recommendations overlap for protein intake. Gray areas denote ranges below or above ISSN recommendations for any fitness volume and intensity

On average, protein intake for all diets met International Society of Sports Nutrition (ISSN) recommendations for general fitness of 0.8-1.2 g/kg/day (Fig. 2) [28]. Carbohydrate intake for most athletes met ISSN recommendations for general fitness of 3-5 g/kg/day on all diets (Fig. 2) [28].

### Training adherence and RPE

Training adherence is summarized in Supplementary Table 2. Participants maintained similar training volume and intensity (gauged by sRPE) across diets. Participants reported average RPE scores of ~ 17 out of 20 (“very hard”) during athletic field tests across all diets (Supplementary Table 3).

### Athletic performance

Athletic performance was not significantly different between plant-based diets (WFPB or PBMA) and Animal for primary outcomes (Table 3). Runners covered a similar distance during the Cooper 12-minute timed run on diets. Mean difference in distance covered between WFPB and Animal was − 23.4 m (95% CI: − 107 to 60.0 m; *p* = 0.54) and − 2.9 m (95% CI: − 119 to 113 m; *p* = 0.96) between PBMA and Animal.

**Table 3:** Athletic field test outcomes and mean differences between diets

Outcome	WFPB	PBMA	Animal	WFPB - Animal	PBMA - Animal
	Mean ± SD^a^	Mean ± SD	Mean ± SD	Mean Difference^b^ 95% CI	Mean Difference 95% CI
**Runners**					
*Primary*					
12-minute timed run, m	2768 ± 347	2789 ± 378	2791 ± 391	− 23.4 (− 107, 60.0)	−2.9 (− 119, 113)
*Secondary*					
VO_2_ max, mL O_2_/kg/min	50.1 ± 5.7	49.6 ± 5.4	48.9 ± 5.9	1.2 (− 0.9, 2.5)	0.7 (− 0.2, 1.7)
**Resistance Trainers**					
*Primary*					
Machine composite strength^c^, total kg and %	298 ± 122	303 ± 123	313 ± 144	−2.7 (− 5.8, 0.4)	−0.7 (− 3.5, 2.2)
*Secondary*					
Push-up, n	34.9 ± 8.8	35.0 ± 7.6	37.6 ± 14.8	−2.7 (− 8.0, 2.5)	−2.6 (− 9.0. 3.8)
Pull-up, n	13.3 ± 3.2	13.5 ± 2.6	13.9 ± 3.0	− 0.6 (− 1.8, 0.5)	−0.5 (− 1.6, 0.7)
Chest press, kg	67 ± 32	67 ± 32	68 ± 32	− 1.2 (− 3.5, 1.1)	−0.6 (− 3.4, 2.2)
Leg press, kg	166 ± 78	170 ± 77	177 ± 99	−11.5 (− 28.0, 4.9)	−7.2 (− 24.0, 9.6)
Lat pull-down, kg	65 ± 22	66 ± 21	68 ± 23	− 2.3 (− 5.7, 1.1)	−1.4 (− 3.8, 0.9)

^a^Mean ± SD was the absolute value of distance covered, score, kg, or repetitions

^b^Mean Difference was the average of individual differences between diets in distance covered, score, average % change across machine exercises, or repetitions

^c^Machine composite strength is expressed as a raw score of all machine exercises (kg) for individual diets. Mean difference for machine exercises is expressed as a mean of the average % change for an individual across exercises: [(Chest WFPB – Chest Animal)/Chest Animal + (Leg WFPB – Leg Animal)/Leg Animal + (Lat WFPB – Lat Animal)/Lat Animal]/3

Resistance trainers had similar raw machine composite strength scores on diets in kg, with a non-significant trend for greater strength on Animal. Mean difference in machine composite strength for WFPB and Animal was − 2.7% (95% CI: − 5.8 to 0.4%; *p* = 0.08) and − 0.7% (95% CI: − 3.5 to 2.2% *p* = 0.62) between PBMA and Animal. Individual responses to diets for primary outcomes are displayed in Supplementary Figs. 1 and 2.

A sensitivity analysis was conducted in which one outlying resistance trainer was excluded (seen in Supplementary Figs. 2 and 4); this not change our findings for machine composite strength for WFPB and Animal or PBMA and Animal significantly (− 1.8%; 95% CI: − 4.5 to 0.9% and − 0.1%; 95% CI: − 3.0 to 2.8%, respectively), or change the significance finding for any secondary outcomes that will be discussed.

Athletic performance was also not significantly different between diets for secondary outcomes (Table 3). Runners reported a similar estimated VO_2_ max on diets, with a non-significant trend for higher VO_2_ max on plant-based diets compared to Animal. Resistance trainers completed more push-ups and pull-ups on Animal when compared to plant-based diets, but these differences are not significant. Machine composite strength components (chest press, leg press, lat pull-down) are displayed as additional secondary outcomes in Table 3 for which no significant changes are observed, despite a trend for increased weight lifted (kg) on leg press on Animal. Individual responses to diets for secondary outcomes are displayed in Supplementary Figs. 3 and 4.

On average, all primary and secondary outcomes for runners and resistance trainers increased from baseline, but no training effect across the 12-week study was observed (Supplementary Fig. 5a and b).

### Anthropometrics

Body weight and body fat percentage were lower, < 1 kg or 1% on average, for several comparisons between plant-based diets and Animal. Runners had significantly lower body weight and body fat on WFPB compared to Animal, with mean differences of − 0.5 kg (95% CI: − 0.8 to − 0.3 kg) and − 0.2% (95% CI: − 0.3 to − 0.1%) respectively. Runners had significantly lower body fat on PBMA compared to Animal, with a mean difference of − 0.2% (95% CI: − 0.4 to − 0.1%). Resistance trainers also saw a significant decrease in body weight of − 0.9 kg (95% CI: − 1.6 to 0 kg) and body fat of − 0.4% (95% CI: − 0.7 to 0%) between WFPB and Animal (Supplementary Table 4).

### Diet satisfaction

Diet satisfaction ratings are presented in Supplemental Fig. 6. WFPB and Animal scored similarly on several categories including taste, appeal, and overall diet satisfaction. PBMA scored lowest on 80% of categories, with notably lower scores for ease of purchase and likeability. Participants also said it required more effort to adhere to PBMA.

## Discussion

*SWAP-MEAT Athlete* explored the impact of two predominately plant-based diets—WFPB and PBMA—compared to an omnivorous diet favoring red meat and poultry (Animal) on endurance and muscular strength. No significant differences were seen in primary (12-minute timed run or machine composite strength) or secondary (estimated VO_2_ max or push-up, pull-ups) athletic performance outcomes for runners or resistance trainers. Our study is one of the first to investigate plant-based meat alternatives and athletic performance, and to our knowledge, this is one of the largest randomized crossover trials (*n* = 22) conducted on plant-based diets and athletic performance.

Our results for runners are consistent with previous studies. Raben et al., 1992 found no difference in endurance performance in a 2 × 6 week crossover study of eight men consuming lacto-ovo-vegetarian and mixed diets [17]. Hietavala et al., 2012 also found no detriment in time to exhaustion or VO2 max on a low-protein vegetarian diet in nine men in a 2 × 4 day crossover trial [18]. Even after 6 months, Blanquaret et al., 2018 found no differences in endurance performance for women randomized to vegetarian (*n* = 15) or omnivorous diets (*n* = 10) [29]. Some cross-sectional studies suggest plant-based diets confer superior endurance performance. Lynch et al., 2016 saw significantly higher VO_2_ max in female vegetarians, but not men [15]. Boutros et al., 2020 observed significantly higher VO_2_ max in vegans (*n* = 28) compared to omnivores (*n* = 28) [16]. In our randomized crossover trial, we observed a trend for increased VO_2_ max on plant-based diets compared to Animal, but this was not significant.

Our results for resistance trainers are consistent with existing studies, most of which are cross-sectional. Hevia-Larrain et al., 2021 found no significant difference in 1-rep max leg press between habitual vegans (*n* = 19) or omnivores (*n* = 19) with plant-based or animal protein supplementation [30]. Lynch et al., 2016 found a trend for greater peak torque during leg extension in omnivores (*n* = 43) compared to vegetarians (*n* = 27), but this difference was not significant [15]. Similarly, Boutros et al., 2020, found no significant differences in strength (1-rep max leg press and seated chest press) in vegan or omnivorous females (*n* = 56), despite observing a modest trend for greater chest press in omnivores [16]. This is consistent with our observation, albeit not significant, of increased machine composite strength on Animal. Existing randomized controlled trials show no significant differences in strength between diets after 12 weeks, but focus on older men and physical function [31–33].

Our randomized crossover trial adds to the current body of literature by investigating athletic performance outcomes in larger, more generalizable sample of recreational male and female athletes across a variety of endurance and strength outcomes and found no significant differences in performance. Additionally, we found that PBMA–a new, emerging form of plant-based diets–did not significantly change performance compared to Animal or more traditional WFPB diets.

### Protein recommendations

Our findings of no significant differences in athletic performance may be related to the fact that adequate amounts of protein were consumed on all diets. On average, participants met ISSN recommendations for protein intake for general fitness on all diets [28]. Notably, average energy intake for our athletes was lower than expected—but no large reductions in body weight were seen—which suggests underreporting of energy and macronutrient intake. This is common in studies on athletes, with energy intake underestimated by 19% on average [34]. Yet, even with underreporting, protein intake for athletes already met or exceeded recommended levels of 0.8-1.2 g/kg/day on all diets.

These findings contradict the popular belief that a predominately plant-based diet does not contain enough protein to support athletic performance and adaptations, and it is possible that protein is overemphasized in athletic spheres. Previous studies show that athletes and their coaches overestimate the amount of protein they need, and many mistakenly believe that protein—rather than carbohydrates—functions as the body’s primary fuel source [35, 36]. In contrast, medical professionals recognize that plant-based diets can provide athletes with both an appropriate quality and quantity of protein [9, 11].

It should be noted that elite athletes may need to consume more protein than ISSN recommendations for general fitness of 0.8-1.2 g/kg/day. However, a recent review by Morton et al., 2018 showed protein intake beyond 1.6 g/kg/day provided no additional increase in lean muscle mass. Many recreational athletes in our study exceeded 1.6 g/kg/day on Animal, even with underreported values, which suggests athletes may not need to overemphasize protein intake from animal meat to meet recommendations [37]. Our dietary intake data also highlights the PBMA diet pattern as an intermediate between Animal and WFPB in protein and macronutrient composition. Athletes with high protein demands could consider supplementing WFPB with protein from PBMA to more easily reach recommendations within a predominately plant-based diet.

### Carbohydrate recommendations

On average, athletes also met ISSN recommendations for carbohydrate intake for general fitness on all diets [28]. Carbohydrates are the body’s primary energy source and are crucial for endurance athletes who rely on glycogen reserves [38]. However, previous literature suggests that many athletes do not consume enough carbohydrates [35]. This can occur when protein is over-prioritized in omnivorous diets. Protein consumption is crucial for muscle recovery and synthesis, but emphasis on protein intake by athletes reaches a point of diminishing return if caloric intake remains unchanged and carbohydrates are swapped for protein [12, 38]. In our study, reported values for carbohydrate intake for some athletes fell below ISSN recommendations on Animal, but this did not impact performance within 4 weeks. In the future, athletes who do not meet ISSN recommendations on an omnivorous diet might benefit from incorporating more plant-based products (WFPB or PBMA) and/or shifting to a plant-based diet to protect against carbohydrate depletion and provide lasting fuel during exercise.

### Feasibility

All participants included in analysis (*n* = 22) completed all athletic field tests. Only two participants did not complete the study, which means participant retention was 92%. Participants had high dietary adherence; training volume and intensity were also consistent which increases our confidence in the study findings, as well as the feasibility of a larger randomized crossover trial. Participants also had high adherence to dietary logging on Cronometer, logging on average 15-16 days per diet phase when only 12 were required.

### Strengths

Our three-way randomized crossover trial recruited athletes across a variety of types and sexes: 12 runners (six male, six female) and 12 resistance trainers (six male, six female). The crossover design allowed participants to serve as their own control, and two runners and two resistance trainers were assigned to each diet order to minimize the impact of diet order on performance. We explored two different types of plant-based diet patterns (WFPB and PBMA), and we also recruited recreational athletes—rather than focusing an elite subset of athletes—which increases the generalizability of our findings. Athletic field tests assessed “real world” performance and incorporation of wearables for measuring VO_2_ max may increase external validity of our findings.

### Limitations

We acknowledge several limitations: diet phases lasting 4 weeks are common in literature, but may not have been long enough for adaptation and observation of changes in performance. A second limitation was the absence of washout phases and secondary baseline measurements before new diets. These factors increase participant burden and study duration, but help to isolate the effects of diets. Third, muscle biopsies and dual-energy X-ray absorptiometry scans could explore glycogen storage, muscle protein synthesis, and body composition as mechanisms by which diet impacts athletic performance. However, these were not assessed due to budget constraints. Lastly, the effect of auxiliary animal product intake (dairy, egg) in diets was not isolated from intake of primary protein sources (animal meat, plant-based meat alternative, whole plant proteins). However, the study was not reductionistic – we intended to study generalizable plant-based diet patterns rather than compare dairy and egg vs. other protein sources.

Our study sample size (*n* = 22) was relatively small, yet larger than most previously published randomized crossover studies. However, this sample size may not have allowed us to detect statistically significant differences in performance. More importantly, future research must establish what constitutes a meaningful difference in athletic performance (i.e., whether it is worthwhile to change one’s diet just to run five meters farther or lift two more kilograms) regardless of whether or not statistical significance is reached in a larger sample of participants.

## Conclusion

Runners and resistance trainers in the present study experienced no significant change in endurance or muscular strength on two predominately plant-based diets (WFPB and PBMA) compared to Animal. WFPB and PBMA excluded animal meat and deemphasized consumption of dairy and egg, but this did not appear to impact performance.

Protein and carbohydrate intake for recreational athletes met ISSN recommendations for general fitness on all three diets, which is consistent with our findings of no significant differences in athletic performance. Protein intake greatly exceeded ISSN recommendations on Animal, suggesting recreational athletes may not need to overemphasize protein intake from animal meat to meet recommendations.

Our study is one of the first to explore the impact of plant-based meat alternatives on athletic performance, and our dietary intake data highlights PBMA as a potential intermediate between Animal and WFPB in protein and macronutrient composition that could sustain performance. Consumption of PBMA could increase protein intake within plant-based diets for athletes with higher protein needs. Our dietary intake data also highlights how plant-based diets (WFPB and PBMA) can increase carbohydrate intake which is essential for athletic performance.

Overall, no significant changes in any athletic performance outcome were seen between diets which suggests that both WFPB and PBMA can serve as a viable option for recreational athletes to adopt. With these findings, recreational athletes can begin to feel more confident that replacing animal meat and shifting to a more plant-based diet will allow them to achieve adequate protein intake and maintain athletic performance.

## Supplementary Information

**Additional file 1: Supplementary Table 1.** Nutrient Profiles per Serving of Whole Food Plant-Based Proteins, Plant-Based Meat Alternatives, and Animal Meat. **Supplementary Table 2.** Weekly Adherence to Consistent Physical Activity Across Diet Phases. **Supplementary Table 3.** Rate of Perceived Exertion (RPE) on Athletic Field Test. **Supplementary Table 4.** Anthropometric Measures after 4-wk diet phases. **Supplementary Fig. 1.** Primary Athletic Field Test Outcome (Runners): 12-Minute Timed Run. **Supplementary Fig. 2.** Primary Athletic Field Test Outcome (Resistance Trainers): Machine Composite Strength. **Supplementary Fig. 3.** Secondary Athletic Field Test Outcome (Runners): VO2 max. **Supplementary Fig. 4a.** Secondary Athletic Field Test Outcome (Resistance Trainers): Push-Up. **Supplementary Fig. 4b.** Secondary Athletic Field Test Outcome (Resistance Trainers): Pull-Up. **Supplementary Fig. 4c.** Secondary Athletic Field Test Outcome (Resistance Trainers): Chest Press. **Supplementary Fig. 4d.** Secondary Athletic Field Test Outcome (Resistance Trainers): Leg Press. **Supplementary Fig. 4e.** Secondary Athletic Field Test Outcome (Resistance Trainers): Lat Pull-Down. **Supplementary Fig. 5a.** Athletic Performance over the 12-week intervention: 12-minute timed run. **Supplementary Fig. 5b.** Athletic Performance over the 12-week intervention: machine composite strength. **Supplementary Fig. 6.** Diet Satisfaction.

## Data Availability

Data described in the manuscript, code book, and analytic code will be made available by the corresponding author, Aubrey K. Roberts, upon reasonable request.

## Abbreviations

Animal: Omnivorous diet
ISSN: International Society for Sports Nutrition
PBMA: Plant-based meat alternative diet
RPE: Rate of perceived exertion
WFPB: Whole food plant-based diet
