# Supplementary material for: SWAP-MEAT Athlete (study with appetizing plant-food, meat eating alternatives trial) – investigating the impact of three different diets on recreational athletic performance: a randomized crossover trial
Source: Nutr J. 2022 Nov 16;21:69. doi: 10.1186/s12937-022-00820-x (PMC9666956; doi:10.1186/s12937-022-00820-x)
Supplement: Supplementary file 1 — Additional file 1: Supplementary Table 1. Nutrient Profiles per Serving of Whole Food Plant-Based Proteins, Plant-Based Meat Alternatives, and Animal Meat. Supplementary Table 2. Weekly Adherence to Consistent Physical Activity Across Diet Phases. Supplementary Table 3. Rate of Perceived Exertion (RPE) on Athletic Field Test. Supplementary Table 4. Anthropometric Measures after 4-wk diet phases. Supplementary Fig. 1. Primary Athletic Field Test Outcome (Runners): 12-Minute Timed Run. Supplementary Fig. 2. Primary Athletic Field Test Outcome (Resistance Trainers): Machine Composite Strength. Supplementary Fig. 3. Secondary Athletic Field Test Outcome (Runners): VO2 max. Supplementary Fig. 4a. Secondary Athletic Field Test Outcome (Resistance Trainers): Push-Up. Supplementary Fig. 4b. Secondary Athletic Field Test Outcome (Resistance Trainers): Pull-Up. Supplementary Fig. 4c. Secondary Athletic Field Test Outcome (Resistance Trainers): Chest Press. Supplementary Fig. 4d. Secondary Athletic Field Test Outcome (Resistance Trainers): Leg Press. Supplementary Fig. 4e. Secondary Athletic Field Test Outcome (Resistance Trainers): Lat Pull-Down. Supplementary Fig. 5a. Athletic Performance over the 12-week intervention: 12-minute timed run. Supplementary Fig. 5b. Athletic Performance over the 12-week intervention: machine composite strength. Supplementary Fig. 6. Diet Satisfaction. [file 12937_2022_820_MOESM1_ESM.docx]

**Supplementary Table 1. Nutrient Profiles per Serving of Whole Food Plant-Based Proteins, Plant-Based Meat Alternatives, and Animal Meat**^1^

| Product | | Serving Size^2^ | Kcals | | Protein,  g | Carbs, g | Total fat, g | Sat fat,  g | Fiber, g | Sodium, mg |
| --- | --- | --- | --- | --- | --- | --- | --- | --- | --- | --- |
| *Whole Food Plant-Based* |  | | |  |  |  |  |  |  |  |
| Tofu^3^ | | 3 oz | | 88 | 10 | 3 | 5 | 1 | 1 | 14 |
| Tempeh | | 3 oz | | 163 | 17 | 6 | 9 | 2 | 0 | 8 |
| Quinoa | | ½ cup | | 250 | 10 | 44 | 4 | 0 | 5 | 3 |
| Black Beans | | ½ cup | | 96 | 6 | 18 | 0 | 0 | 7 | 265 |
| *Plant-Based Meat Alternatives*  Impossible Burger | | 4 oz | | 240 | 19 | 9 | 14 | 8 | 3 | 370 |
| Beyond Beef Ground | | 4 oz | | 230 | 20 | 7 | 14 | 5 | 2 | 390 |
| Gardein Chick’n Strips | | 4 oz | | 164 | 18 | 6 | 8 | 1 | 0 | 387 |
| *Animal Meat* ^4^ | |  | |  |  |  |  |  |  |  |
| Burger | | 3 oz | | 216 | 21 | 0 | 14 | 5 | 0 | 57 |
| Pork | | 3 oz | | 214 | 23 | 0 | 13 | 5 | 0 | 41 |
| Chicken Breast | | 3 oz | | 147 | 26 | 0 | 4 | 1 | 0 | 65 |

^1^All nutrient profiles obtained from NDSR Software Food and Nutrient Database.

Dairy and egg were minimized in WFPB, and consumption was not emphasized and did not count as a primary protein source in PBMA or Animal.

^2^4 oz of PBMA was chosen to more closely achieve caloric and protein equivalence with 3 oz of Animal

^3^Tofu is minimally processed and thus considered WFPB rather than PBMA

^4^Animal emphasized protein intake from red meat and poultry. Fish limited to once per week.

**Supplementary Table 2. Weekly Adherence to Consistent Physical Activity Across Diet Phases**

| Outcome | WFPB | | |  | | PBMA | Animal | |
| --- | --- | --- | --- | --- | --- | --- | --- | --- |
|  | Median | IQR |  | | Median | IQR | Median | IQR |
| **Runners**  *Volume* |  |  |  | |  |  |  |  |
| Minutes of running | 157 | (97 - 213) |  | | 161 | (111 - 241) | 139 | (115 - 228) |
| Minutes of other activity | 123 | (91 - 153) |  | | 132 | (87 - 188) | 105 | (83 - 159) |
| Miles per week | 18.9 | (11.3 - 23.1) |  | | 19.0 | (9.5 - 25.8) | 17.0 | (10.7 - 24.0) |
|  |  |  |  | |  |  |  |  |
| *Intensity*    Average sRPE^1^ | 12.4 | (11.3 - 13) |  | | 12.3 | (11.4 -12.7) | 12.5 | (11.3 - 13) |
|  |  |  |  | |  |  |  |  |
| **Resistance Trainers**  *Volume* |  |  |  | |  |  |  |  |
| Minutes of resistance training | 206 | (156 - 256) |  | | 165 | (133 - 248) | 203 | (123 - 259) |
| Minutes of other activity^2^ | 114 | (84 - 190) |  | | 188 | (146 - 341) | 131 | (75 - 202) |
|  |  |  |  | |  |  |  |  |
| *Intensity*    Average sRPE | 14.8 | (12.9 – 15.5) |  | | 14.8 | (13.1 - 16.3) | 14.8 | (13.1 - 15.9) |

^1^sRPE refers to “session RPE,” the training intensity of individual workouts throughout each diet rated on the 6-20 Borg RPE scale

^2^Other activity included cycling, climbing, hiking, or other cross-training activities for the purpose of exercise

**Supplementary Table 3. Rate of Perceived Exertion (RPE) on Athletic Field Test**

| Outcome | WFPB | PBMA | Animal | WFPB - Animal | PBMA - Animal |
| --- | --- | --- | --- | --- | --- |
|  | Mean ± SD | Mean ± SD | Mean ± SD | Mean Difference^1^,  95% CI | Mean Difference, 95% CI |
| **Runners** |  |  |  |  |  |
| 12 minute timed run | 17.3 ± 3.1 | 16.9 ± 2.5 | 17.5 ± 2.6 | -0.3 (-0.8, 0.3) | -0.6 (-1.5, 0.2) |
| **Resistance Trainers** |  |  |  |  |  |
| Chest press | 16.5 ± 2.9 | 17.4 ± 2.7 | 17.6 ± 2.2 | -1.1 (-2.2, 0) | -1.3 (-1.3, 0.7) |
| Leg press | 16.7 ± 2.8 | 17.3 ± 2.5 | 17.4 ± 2.1 | -0.6 (-1.8, 0.6) | -0.1 (-1.0, 0.8) |
| Lat pull-down | 17.1 ± 2.7 | 17.2 ± 2.6 | 17.5 ± 2 | -0.4 (-1.7, 1.0) | -0.3 (-1.5, 0.9) |
| Push-up | 16.1 ± 3.3 | 17.2 ± 2.4 | 17 ± 2.6 | -0.9 (-1.9, 0.1) | 0.2 (-0.5, 0.9) |
| Pull-up | 16.7 ± 2.9 | 16.9 ± 2.6 | 17 ± 2.5 | -0.3 (-1.4, 0.8) | -0.1 (-1.0, 0.8) |

^1^Mean Difference was the average of individual differences (WFPB - Animal or PBMA - Animal) between diets

**Supplementary Table 4. Anthropometric Measures after 4-wk diet phases**

| Outcome | WFPB  Mean ± SD | PBMA  Mean ± SD | Animal  Mean ± SD | WFPB - Animal  Mean Difference,^1^  95% CI | PBMA - Animal  Mean Difference, 95% CI |
| --- | --- | --- | --- | --- | --- |
|  |  |  |  |  |  |
| **Runners** |  |  |  |  |  |
| Body weight, kg | 63.5 ± 8.2 | 63.5 ± 8.2 | 64 ± 8.2 | -0.5 (-0.8, -0.3) | -0.3 (-0.8, 0.3) |
| Body fat, % | 14.8 ± 3.8 | 14.8 ± 3.7 | 15.0 ± 3.7 | -0.2 (-0.3, -0.1) | -0.2 (-0.4, -0.1) |
| **Resistance Trainers** |  |  |  |  |  |
| Body weight, kg | 64 ± 12.7 | 64.9 ± 13.2 | 64.9 ± 13.2 | -0.9 (-1.6, 0) | 0 (-0.5, 0.5) |
| Body fat, % | 14.7 ± 4.0 | 15.1 ± 4.3 | 15.1 ± 4.3 | -0.4 (-0.7, 0) | 0.1 (-0.1, 0.2) |

^1^Mean Difference was the average of individual differences (WFPB - Animal or PBMA - Animal) between diets

**
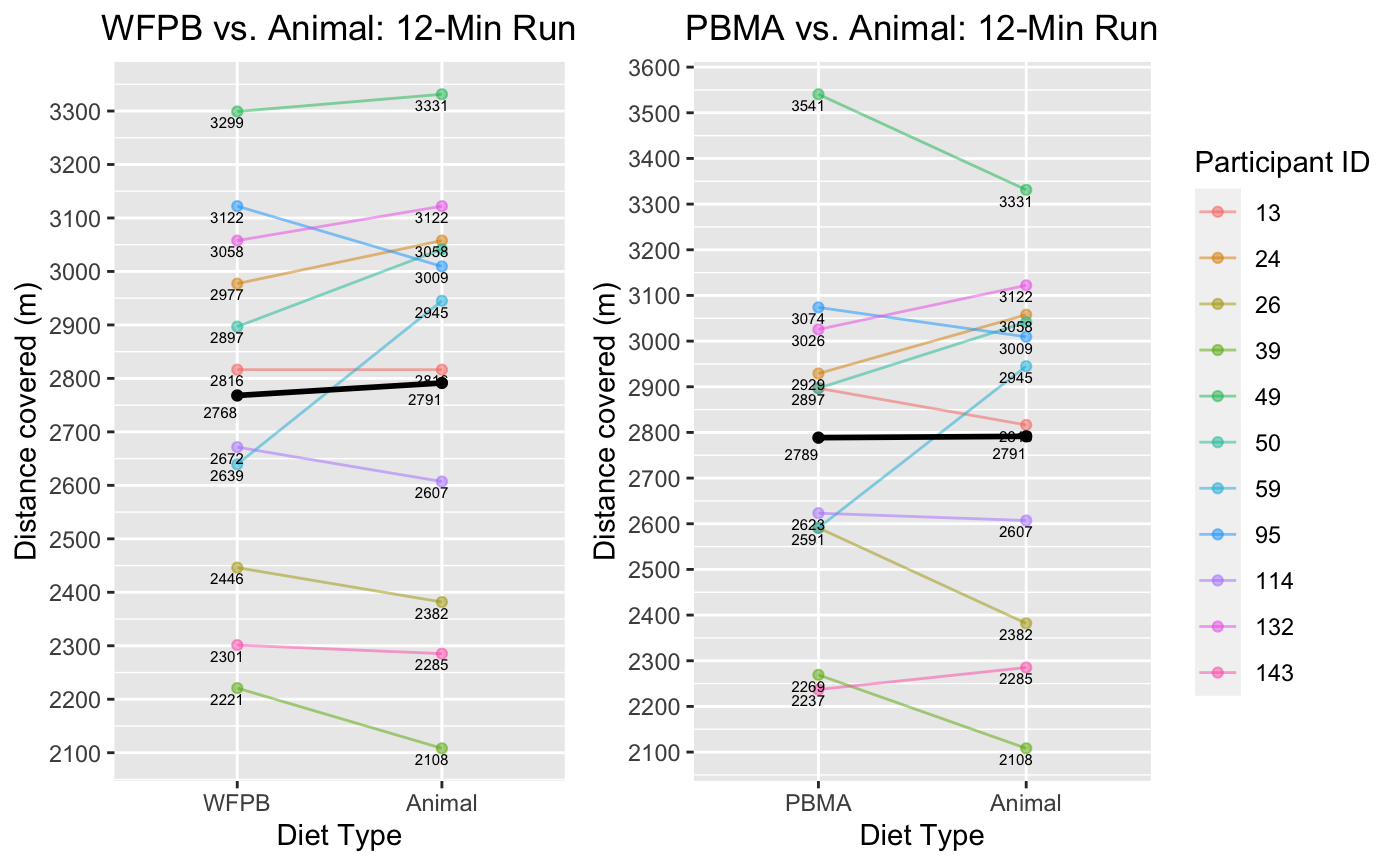
**

**Supplementary Figure 1. Primary Athletic Field Test Outcome (Runners):**

**12-Minute Timed Run**


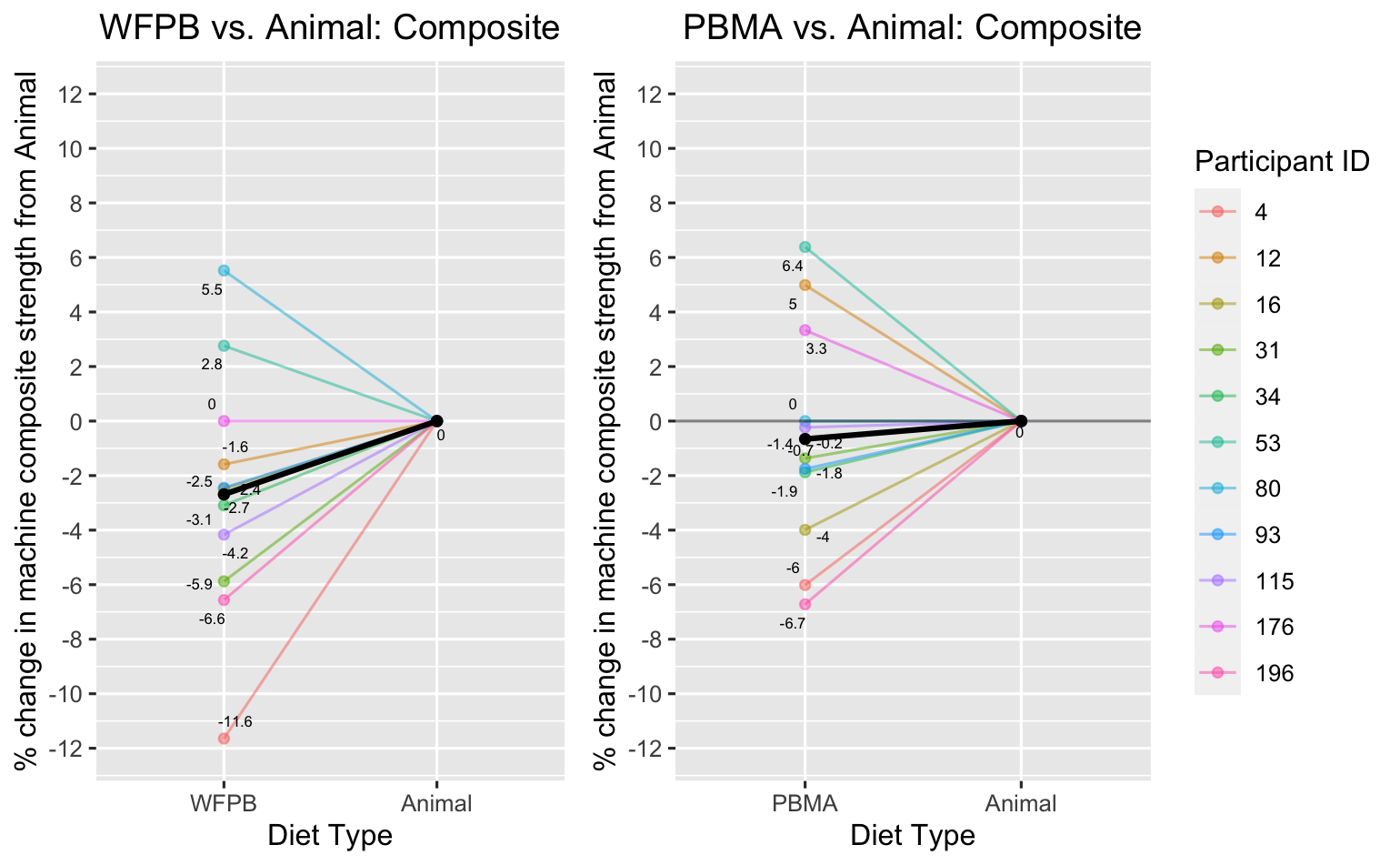


**Supplementary Figure 2. Primary Athletic Field Test Outcome (Resistance Trainers): Machine Composite Strength**


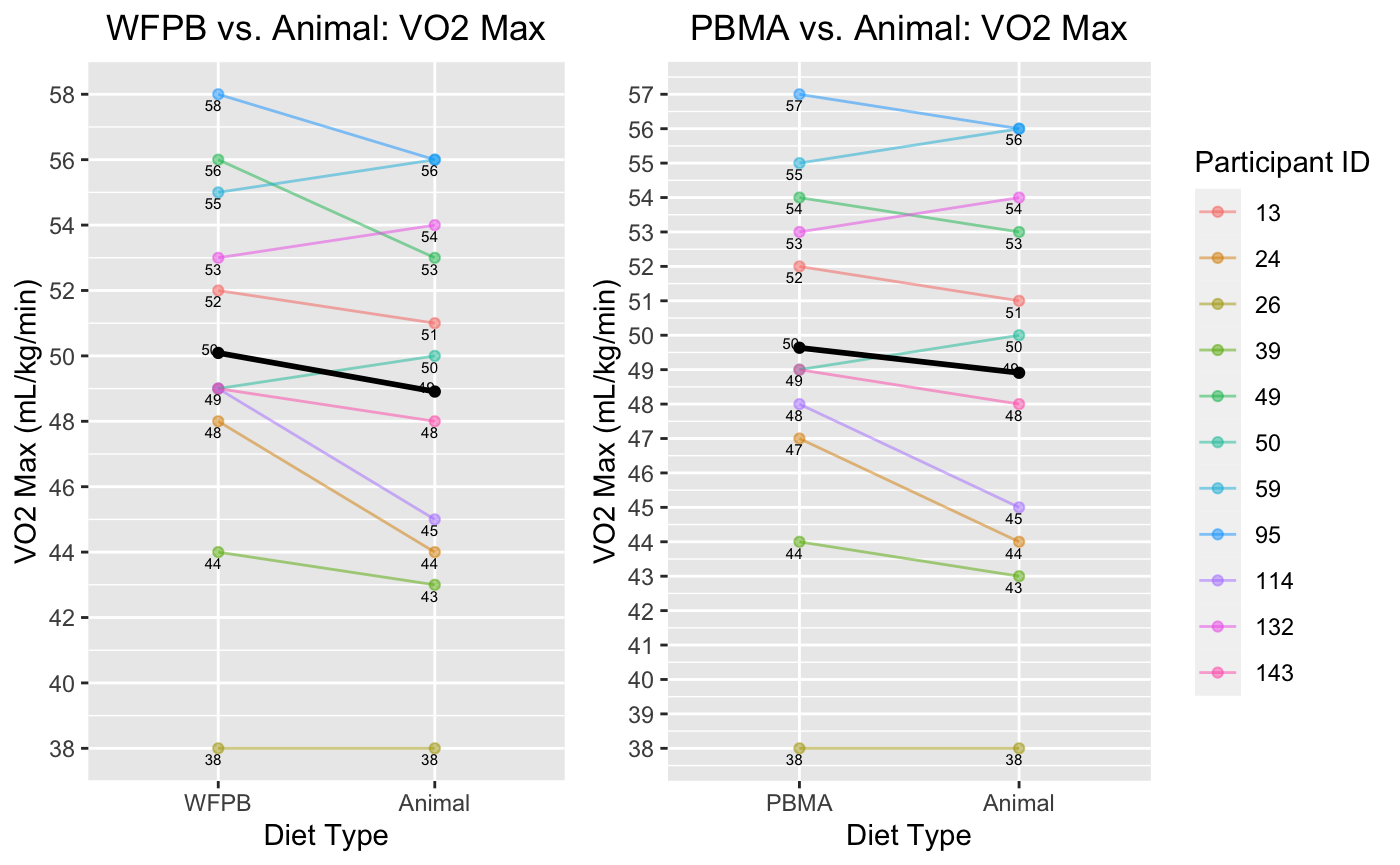


**Supplementary Figure 3. Secondary Athletic Field Test Outcome (Runners):**

**VO_2_ Max**


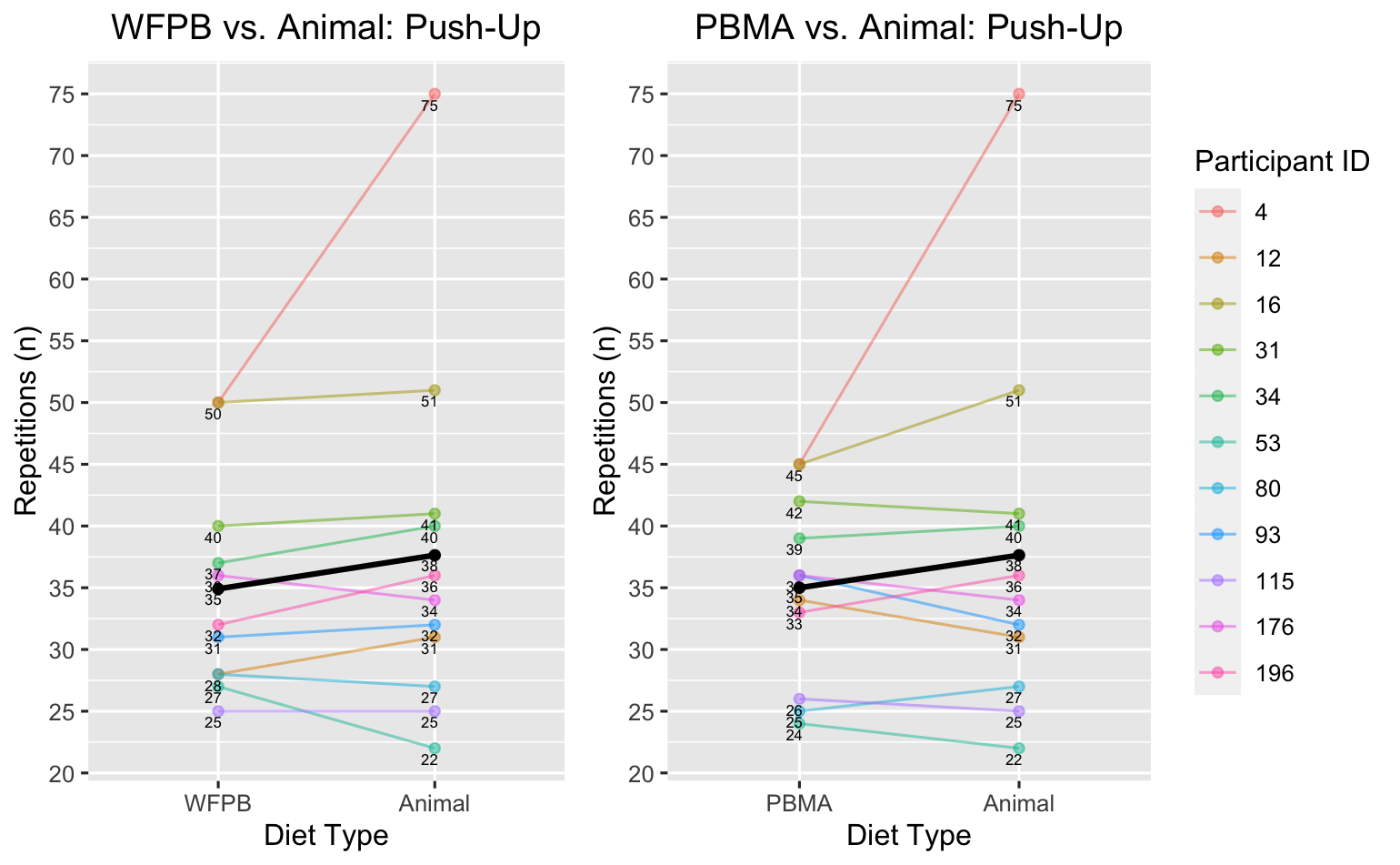


**Supplementary Figure 4a. Secondary Athletic Field Test Outcome (Resistance Trainers): Push-Up**


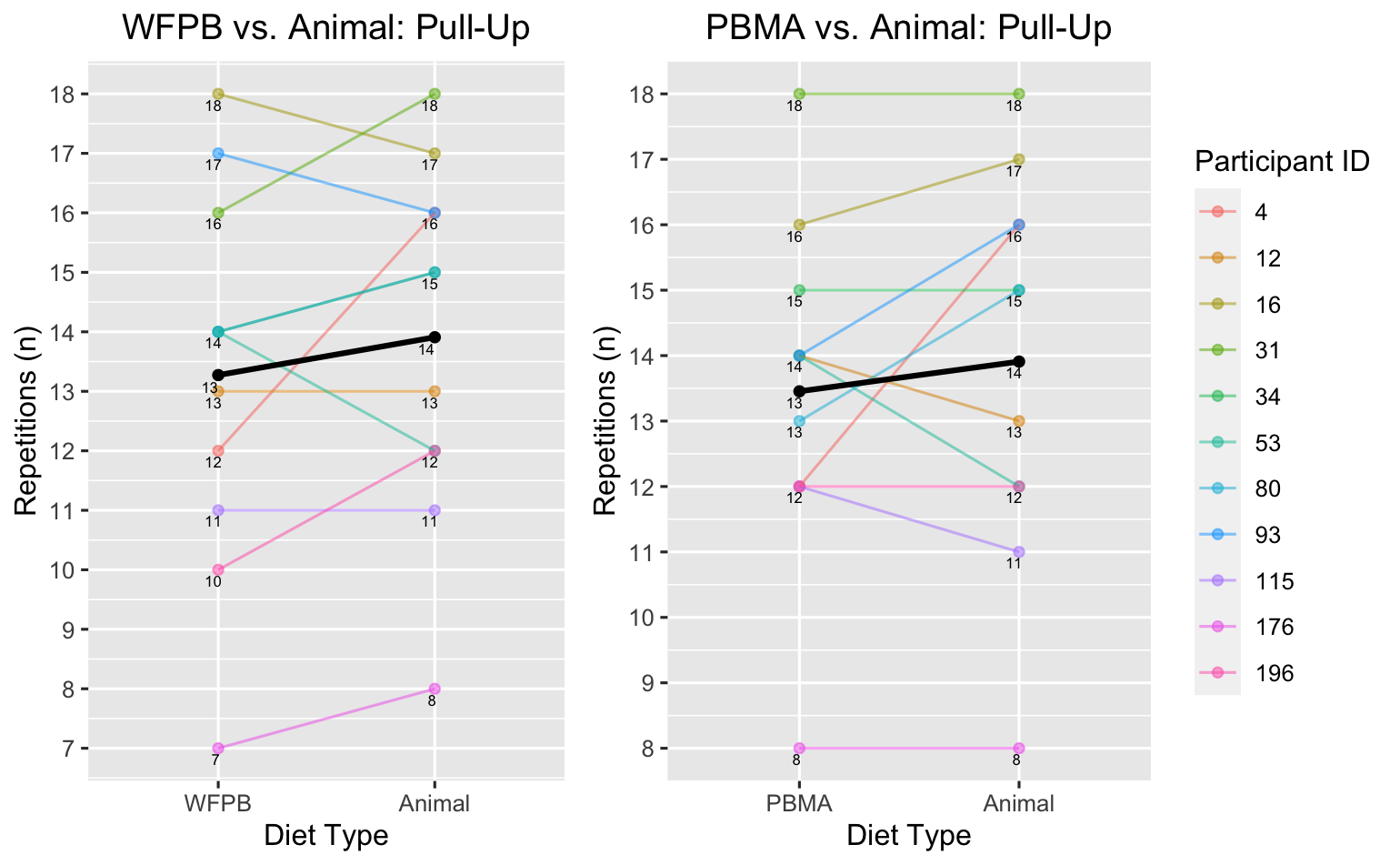
**Supplementary Figure 4b. Secondary Athletic Field Test Outcome (Resistance Trainers): Pull-Up**


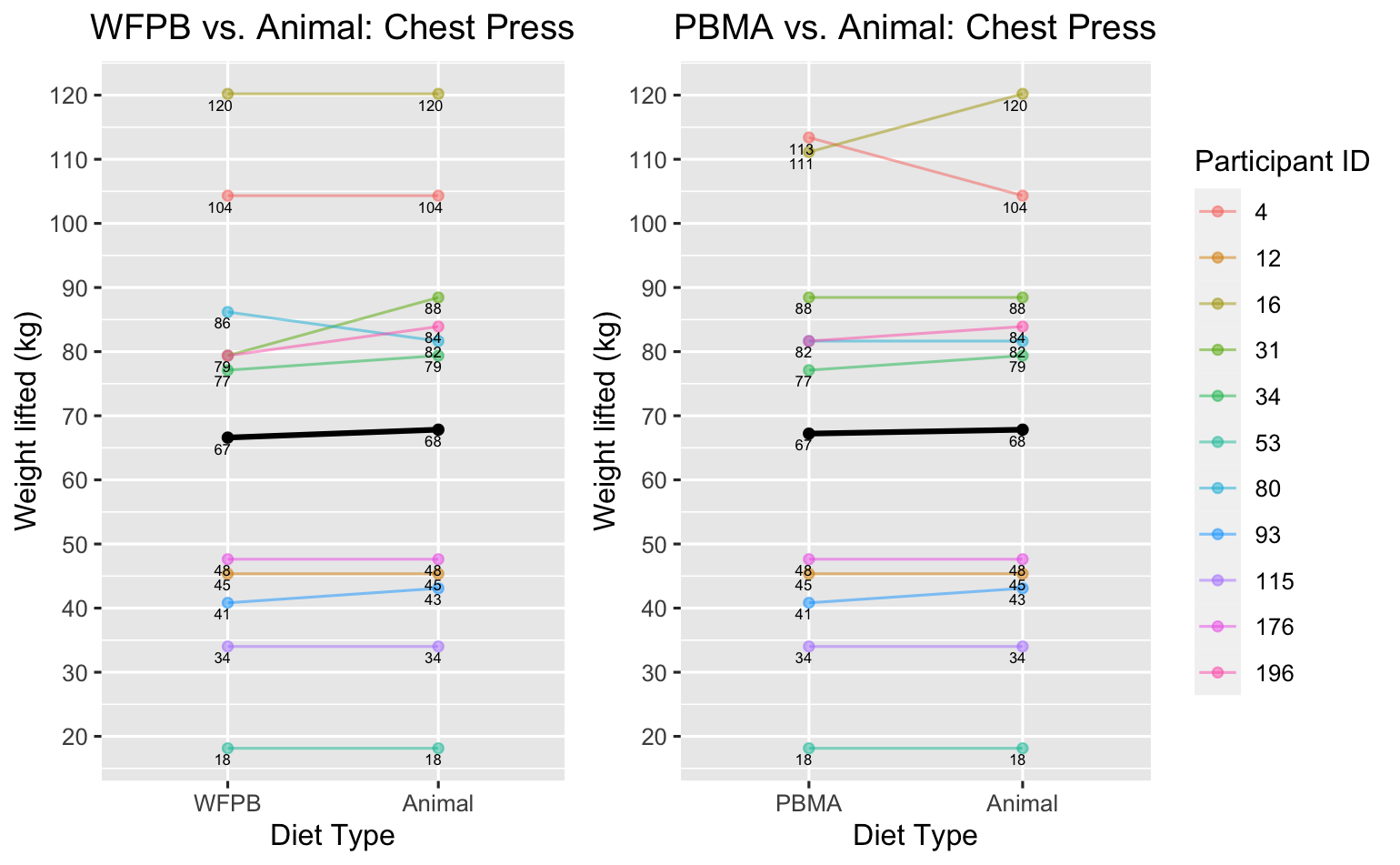


**Supplementary Figure 4c. Secondary Athletic Field Test Outcome (Resistance Trainers): Chest Press**

**
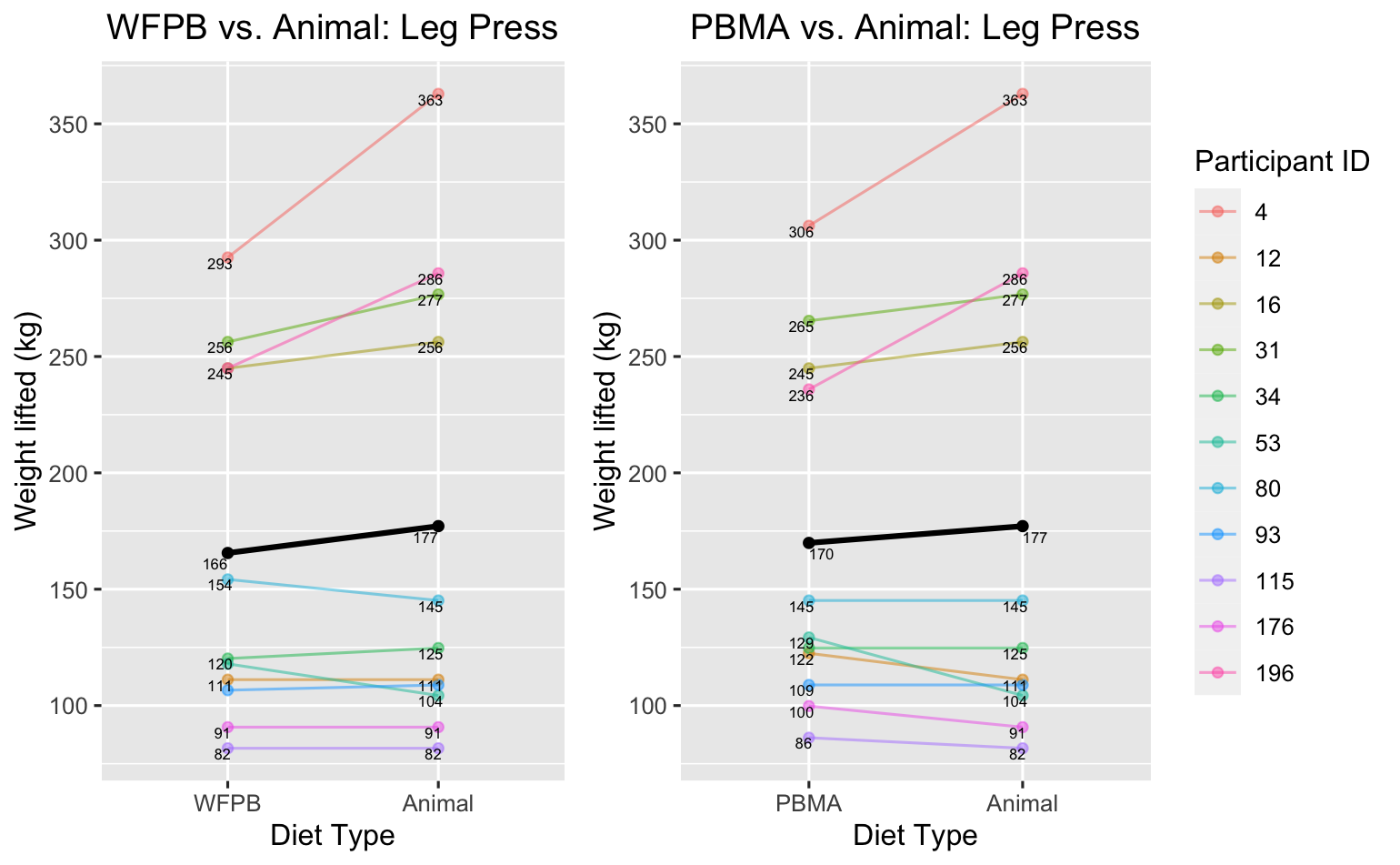
**

**Supplementary Figure 4d. Secondary Athletic Field Test Outcome (Resistance Trainers): Leg Press**

**
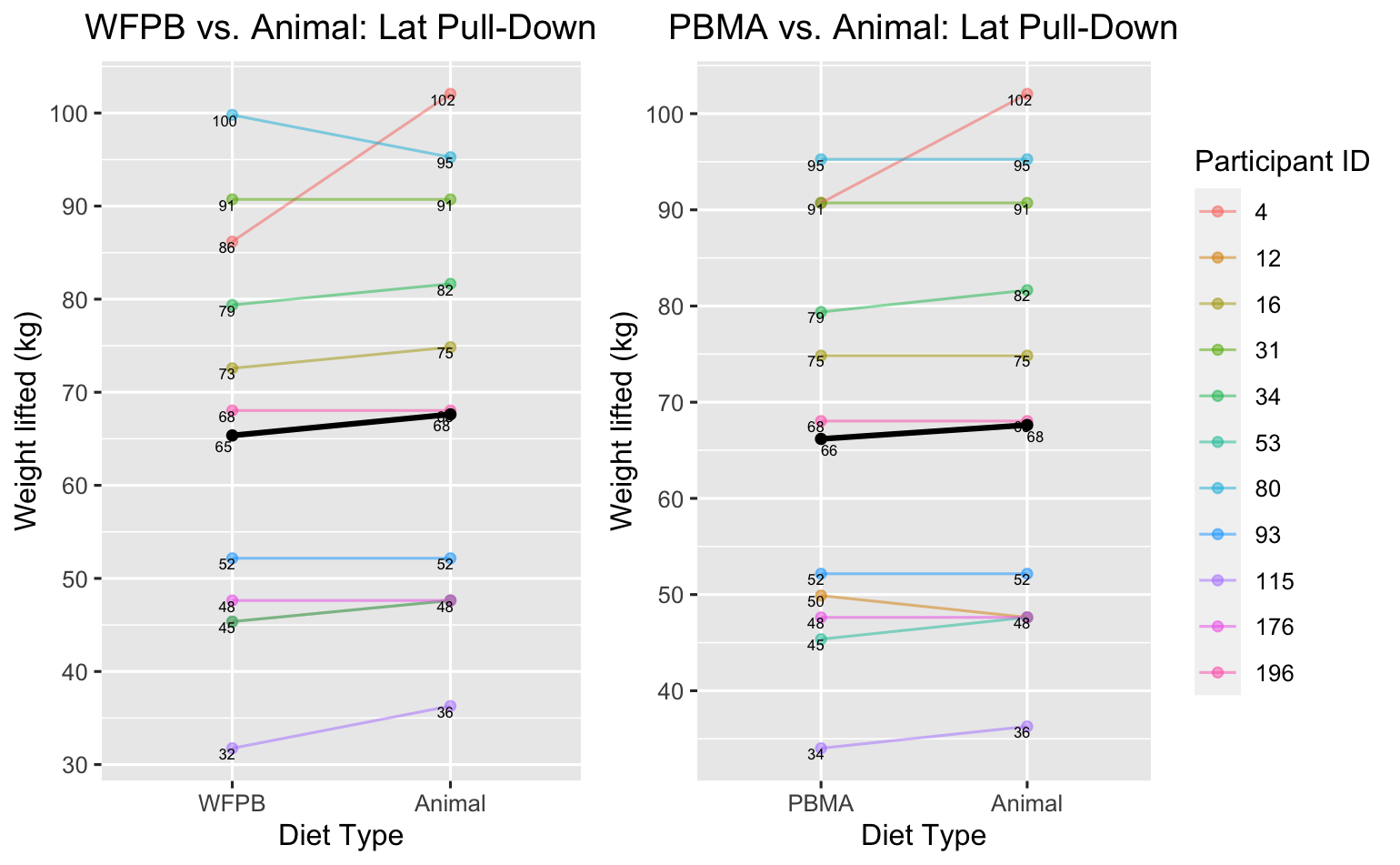
**

**Supplementary Figure 4e. Secondary Athletic Field Test Outcome (Resistance Trainers): Lat Pull-Down**


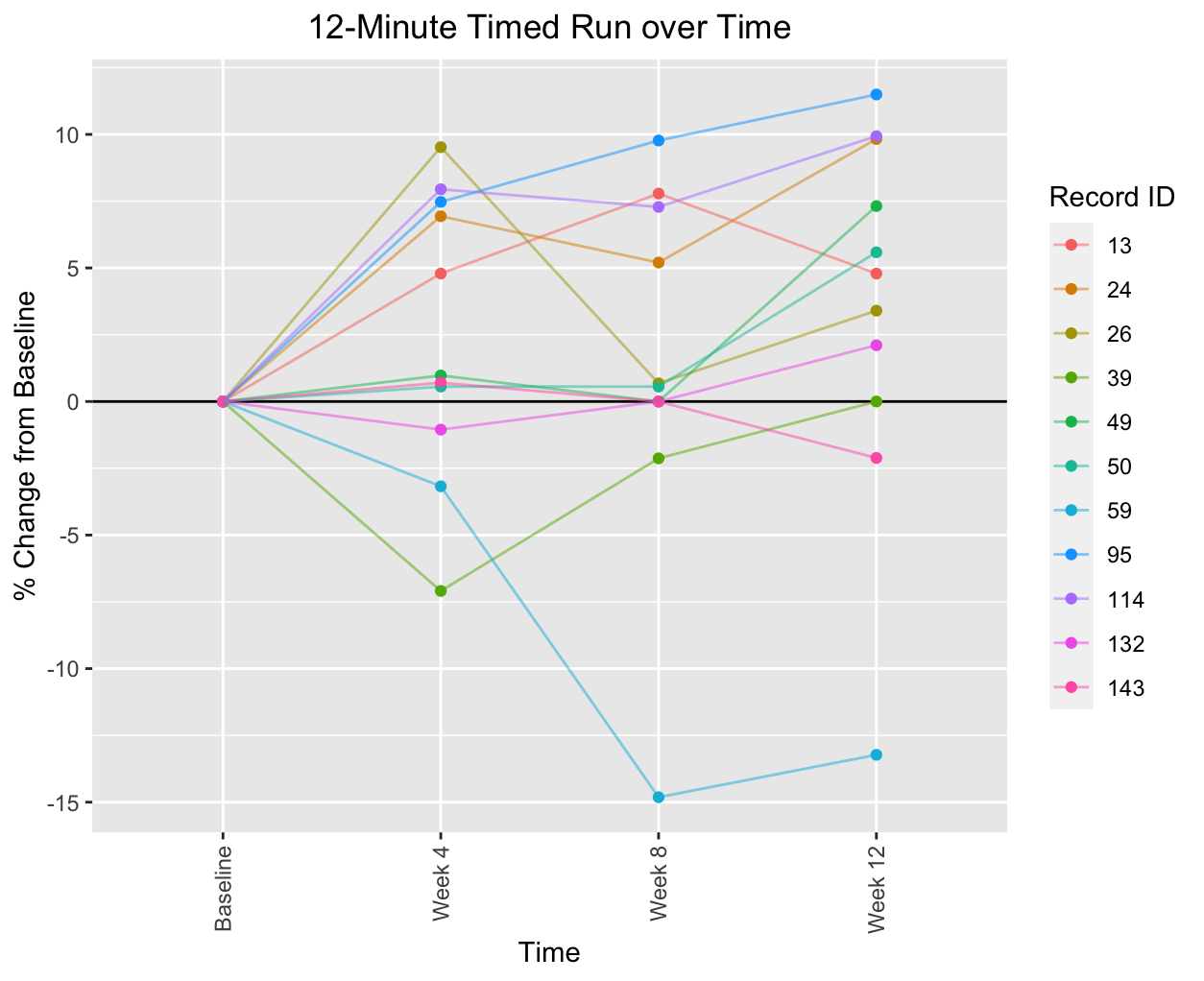


**Supplementary Figure 5a. Athletic Performance over the 12-week intervention: 12-minute timed run**


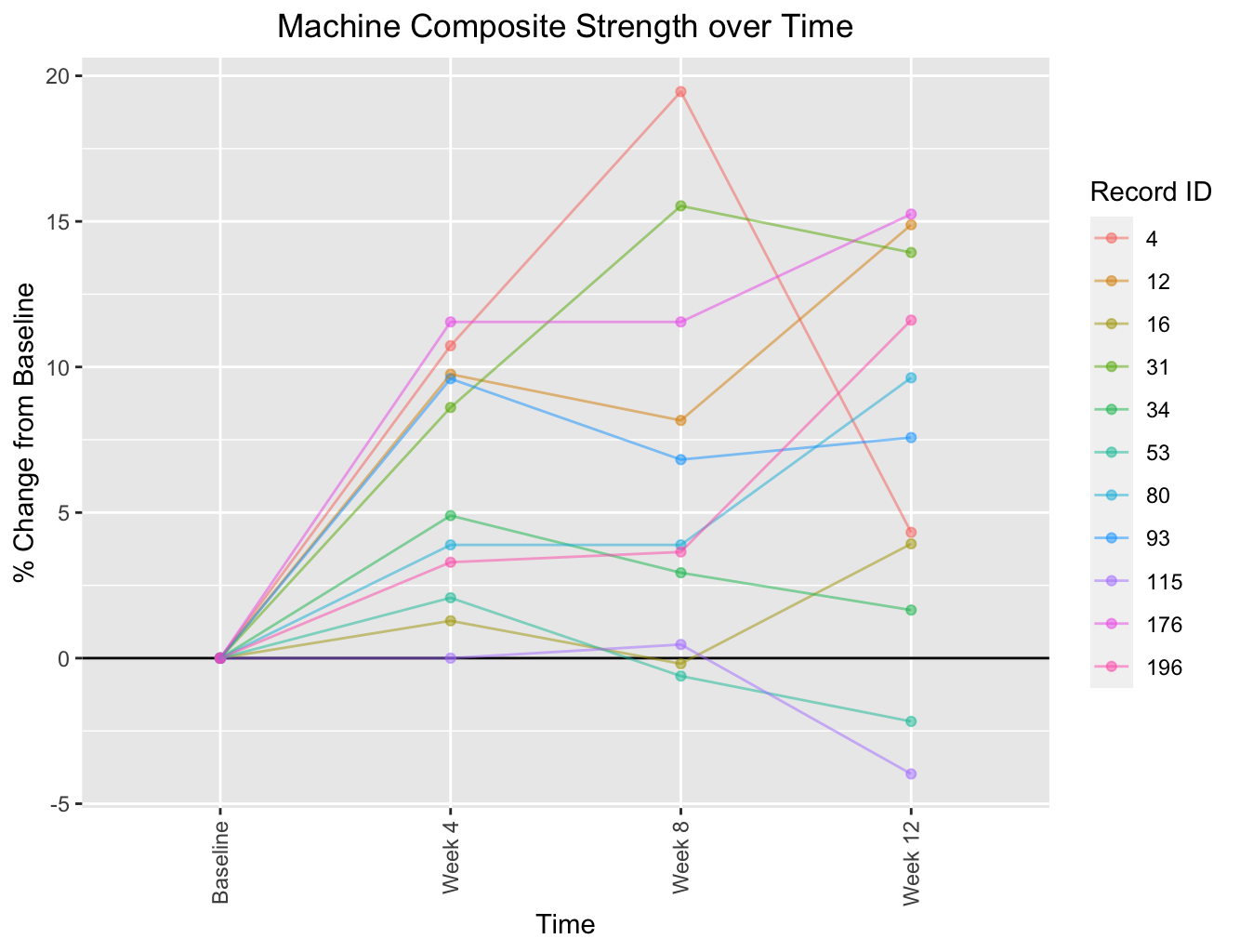


**Supplementary Figure 5b. Athletic Performance over the 12-week intervention: machine composite strength**


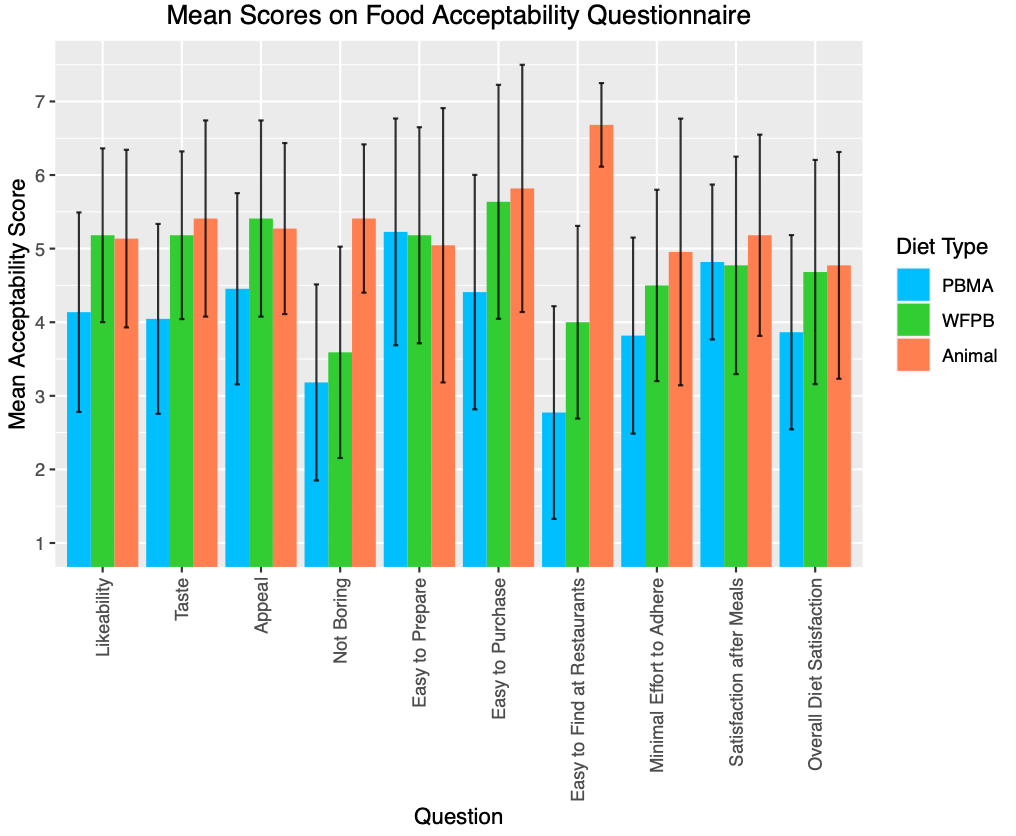


**Supplementary Figure 6. Diet Satisfaction**

Error bars indicate mean ± standard deviation
